# Susceptibility to Hepatitis B Virus Infection Among People Who Inject Drugs in Montreal, Canada

**DOI:** 10.1111/dar.14091

**Published:** 2025-06-03

**Authors:** Olivia Price, Sarah Larney, Valerie Martel‐Laferrière, Julie Bruneau, Brendan Harney

**Affiliations:** ^1^ National Drug and Alcohol Research Centre UNSW Sydney Sydney Australia; ^2^ Centre de Recherche du Centre Hospitalier de L'université de Montréal Montréal Canada; ^3^ Department of Family Medicine and Emergency Medicine, Faculty of Medicine University of Montréal Montréal Canada; ^4^ Department of Microbiology, Infectious Diseases and Immunology, Faculty of Medicine University of Montréal Montréal Canada

## Abstract

**Introduction:**

People who inject drugs are at elevated risk of hepatitis B virus (HBV) infection, which is preventable by vaccination. We examined susceptibility to HBV infection among a sample of people who inject drugs and live in Montreal, Canada.

**Methods:**

Data were obtained from HEPCO, a prospective cohort study of people who had recently (within the past six months) injected drugs, between November 2022 and March 2024. The absence of hepatitis B surface antibody indicated susceptibility to HBV infection. These results were compared to self‐report immune status. Logistic regression was used to identify factors associated with HBV susceptibility.

**Results:**

Overall, 28.1% (108/384) of participants were susceptible to HBV infection. Over half (60.2%, 231/384) of participants correctly reported their immune status. Individuals born in Canada prior to the introduction of universal childhood vaccination programs had higher odds of susceptibility to HBV infection (adjusted odds ratio: 2.63, 95% confidence interval: 1.34–5.61), while those in opioid agonist treatment (0.60, 0.37–0.98) and with a history of hepatitis C infection (0.51, 0.32–0.83) had lower odds of HBV susceptibility.

**Discussion and Conclusions:**

An important minority of people who inject drugs in Montreal remain susceptible to HBV infection. Moderate concordance between self‐report and serological results indicates that serology or vaccine registry information should continue to be used to inform immunisation provision. People who inject drugs who were born prior to childhood vaccination programs and/or are not in opioid agonist treatment are subpopulations who require targeted interventions to increase vaccination coverage.

## 1 Introduction

Although universal childhood vaccination programs have reduced hepatitis B incidence globally, prevalence among people who inject drugs remains high [1]. Hepatitis C prevalence among this population is also high [2], increasing the risk of hepatitis B/C co‐infection, which is associated with more severe negative health outcomes [3]. Maximising vaccination coverage among this population is crucial to achieve the World Health Organization's goal to reduce new hepatitis B infections by 90% and deaths by 65% between 2016 and 2030.

Hepatitis B vaccination is recommended for people who inject drugs in the Canadian Immunisation Guide [4] and funded for this population in Québec [5]. However, people who inject drugs typically have suboptimal vaccination coverage [6]. Barriers to vaccination differ by vaccine and setting, but a recent US paper described competing priorities (e.g., locating stable housing) and the need for multiple vaccine doses as barriers to hepatitis B vaccination among this population [7]. Injecting drug use was associated with acute hepatitis B infections diagnosed between 1990 and 2015 in British Columbia, suggesting that current vaccination coverage is insufficient to completely prevent transmission among this population [8].

Measuring the proportion of a population who remain susceptible to HBV infection can provide important information to policymakers about the effectiveness of a vaccination program. In 2005, 96% of a sample of people who inject drugs in Montreal reported hepatitis B vaccination [9]. However, self‐report has poor validity [10] and HBV infection confers immunity against further infection, so the vaccinated population is a subset of the immune population. This is a key consideration for a population in which transmission is known to occur. Serology can be used to measure both vaccine‐ and infection‐induced immunity and mitigates issues associated with self‐report.

In this study, we used both serological and survey data from a cohort of people who inject drugs in Montreal to: (i) determine susceptibility to hepatitis B virus; (ii) compare immune status using serological data to self‐reported vaccination and infection; and (iii) identify sociodemographic factors associated with susceptibility.

## 2 Methods

### 2.1 Setting and Study Design

Data came from HEPCO, a prospective cohort study established in 2004 that follows people who actively inject drugs in Montreal, Canada [11]. Participants are recruited via a combination of street‐level strategies and community referral. People are eligible for the study if they are aged ≥ 18 years, report injecting drug use in the six months prior to screening, and reside in the Montreal area. Participants at risk of primary or recurrent hepatitis C virus infection are followed up every three months; those with chronic hepatitis C virus infection are followed up annually. Participants who report ≥ 24 consecutive months of no injecting drug use become ineligible for follow‐up as they are no longer considered part of the target population (people actively injecting drugs).

Hepatitis B surface antibody (HBsAb) has been measured among HEPCO participants since November 2022. Participants whose serology indicated they were susceptible to infection were vaccinated by research staff or referred to immunisation services. This was a cross‐sectional study using data from participants' first interview between 29 November 2022 and 22 March 2024 in which hepatitis B serology results were available.

### 2.2 Ethical Approval

Ethical clearance for the study was obtained through the institutional review board of the Centre de recherche du Centre hospitalier de l'Université de Montréal. Participants provided informed consent at enrolment and were remunerated 40 CAD for their time.

### 2.3 Measures

For aim 1, HBsAb was measured in participants' venous blood samples. Individuals with a titre ≥ 10 mIU/mL were considered immune and therefore not susceptible to hepatitis B. Those with a nonreactive titre (< 10 mIU/mL) were considered susceptible to infection. HBsAb results do not distinguish between vaccination‐ and infection‐induced immunity.

For aim 2, we used participants' responses to survey questions on prior vaccination (receipt of either standard hepatitis B vaccination or combination hepatitis A/B vaccination) and hepatitis B diagnosis to assess concordance between self‐reported and serologically confirmed susceptibility to HBV. We constructed a four‐level self‐report indicator for HBV susceptibility: (i) unknown (responded ‘don't know’ regarding both vaccination and infection or ‘don't know’ to one and ‘no’ to the other); (ii) susceptible (neither vaccinated nor infected); (iii) immune due to infection (including people who reported both infection and vaccination); and (iv) immune due to vaccination (any number of doses).

For aim 3, we analysed the association between HBV susceptibility and the following sociodemographic characteristics. We considered eligibility for the Québec universal childhood vaccination program [4], with participants considered either eligible (born in Canada in 1985 or later or moved to Canada in 1985 or later at an age they were still eligible for vaccination); ineligible because they were born in Canada prior to 1985; or ineligible because they were born outside of Canada. The decision to split the ineligible group by country of birth was motivated by the higher prevalence of hepatitis B infection in foreign‐born Canadian residents [8], although some participants born outside of Canada might have been eligible for vaccination in their country of birth. Participants reported the ethnic group they most identified with, which was categorised as White; Aboriginal, Inuit or Métis; or other. Small numbers necessitated the latter umbrella category. Participants also reported their gender identity (woman/female; man/male; non‐binary/queer/other), recent (i.e., past three months) accommodation (stable [living in own/friend's/family's house/apartment, mid/long term accommodation]; unstable [prison, hotel/motel, boarding house, health facility, psychiatric facility], or street/shelter), recent (i.e., past three months) opioid agonist treatment (OAT; yes/no), and incarceration (never; yes > three months ago; yes ≤ three months ago). The latter two variables were included as they are settings where vaccination or referral to vaccination is known to occur [12, 13, 14]. Participants reported which drugs they had ever injected and the age at which they first injected them. This information was used to ascertain the duration since injecting drug use initiation (categorised as ≤ 5 years and > 5 years). Hepatitis C antibody, indicative of lifetime exposure to hepatitis C virus, was detected using enzyme immunoassay.

### 2.4 Analysis

We assessed the association between hepatitis B susceptibility and sociodemographic and health characteristics using the Chi‐squared test. Variables associated with the outcome at *p* < 0.05 were included in a multivariable logistic regression model. Concordance between self‐report and serology immune status was reported descriptively.

Analyses were conducted in R (version 4.3.1).

## 3 Results

Since November 2022, 418 participants completed at least one interview. Of these, 91.9% (384/418) had valid hepatitis B serology data and were included for analysis. Sociodemographic and health characteristics were similar between included and excluded participants, except more men were retained for analysis (Table A1). Among the sample for analysis, most identified as men (82.3%), were ineligible for the Canadian childhood hepatitis B vaccination (i.e., born prior to 1985 or born outside of Canada; 80.5%), and first injected drugs more than five years ago (85.9%; Table 1). Almost half had recently been prescribed OAT (44.5%).

**TABLE 1:** Susceptibility to hepatitis B virus.

	All participants, *n* (%)	% susceptible to hepatitis B virus, (*n*/*N*)	*p*	aOR (95% CI)^a^
Total	384 (100.0)	28.1% (108/384)	—	—
Eligibility for Canadian universal childhood vaccination program				
Eligible	72 (18.8)	15.3% (11/72)	0.023	1
Ineligible, born in Canada prior to 1985	286 (74.5)	31.1% (89/286)		2.63 (1.34, 5.61)
Ineligible, born outside of Canada	23 (6.0)	30.4% (7/23)		1.66 (0.50, 5.24)
Missing	3 (0.8)			
Gender				
Man/male	316 (82.3)	29.1% (92/316)	0.691	/
Woman/female	57 (14.8)	24.6% (14/57)		
Nonbinary, queer or other gender identity	7 (1.8)	14.3% (1/7)		
Missing	4 (1.0)			
Ethnicity				
White	335 (87.2)	29.0% (97/335)	0.409	/
Aboriginal, Inuit or Métis	16 (4.2)	12.5% (2/16)		
Other	33 (8.6)	27.3% (9/33)		
Missing	0 (0)			
Housing^b^				
Stable	205 (53.4)	25.9% (53/205)	0.551	/
Unstable	24 (6.2)	29.2% (7/24)		
Street or shelter	154 (40.1)	31.2% (48/154)		
Missing	1 (0.3)			
Incarceration				
Never	106 (27.6)	34.0% (36/106)	0.212	/
Yes, > 3 months ago	242 (63.0)	26.0% (63/242)		
Yes, ≤ 3 months ago	33 (8.6)	21.2% (7/33)		
Missing	3 (0.8)			
Opioid agonist treatment^b^				
No	210 (54.7)	34.3% (72/210)	0.005	1
Yes	171 (44.5)	21.1% (36/171)		0.58 (0.35, 0.94)
Missing	3 (0.8)			
Hepatitis C antibody status				
Negative	155 (40.4)	35.5% (55/155)	0.005	1
Positive	222 (57.8)	22.1% (49/222)		0.50 (0.30, 0.81)
Missing	7 (1.8)			
Duration of injecting drug use				
≤ 5 years	52 (13.5)	32.7% (17/52)	0.508	/
	330 (85.9)	27.6% (91/330)		
Missing	2 (0.5)			

Abbreviations: aOR, adjusted odds ratio; CI, confidence interval.

^a^ Variables associated with the outcome at *p* < 0.05 were included in the multivariable model.

^b^ In the past 3 months.

In total, 28.1% (108/384) were susceptible to hepatitis B virus infection. Overall, 60.2% (231/384) of participants had concordant self‐reported and serological immune status (Table 2). After excluding those who said they did not know, 73.8% (231/313) had concordant results. The majority of those who reported being vaccinated (80.0%, 191/240) or infected (73.3%, 11/15) had serological evidence for hepatitis B immunity. Half of those who reported neither vaccination nor infection were susceptible to infection (50.0%, 29/58).

**TABLE 2:** Concordance between serology (HBsAb) and self‐report vaccination and infection.

		Serology		Total
		Immune	Susceptible	
*Self‐report*	Immune due to vaccination^a^	**191**	49	240
	Immune due to infection^b^	**11**	4	15
	Susceptible (neither vaccinated nor infected)	29	**29**	58
	Unknown^c^	38	21	59
	Missing	7	5	12
	Total	276	108	384

*Note*: Concordant results are bolded.

^a^ Includes individuals who reported being vaccinated with the standard hepatitis B vaccine and/or combination hepatitis A/B vaccine.

^b^ Includes 9 individuals who reported being both infected and vaccinated.

^c^ Includes individuals who responded ‘don't know’ to infection and vaccination questions and those who responded ‘don't know’ to one and ‘no’ to the alternate.

Participants born in Canada prior to the introduction of vaccination programs had higher odds of HBV susceptibility compared to those born since programes were introduced (adjusted odds ratio: 2.63, 95% confidence interval: 1.34–5.61; Table 1). Participants recently prescribed OAT (0.58, 0.35–0.94) and hepatitis C antibody positive (0.50, 0.30–0.81) had lower odds of HBV susceptibility.

## 4 Discussion

Among this sample of people who inject drugs in Montreal, approximately one third were susceptible to HBV infection. This appears to be similar to the Canadian adult population (30% not vaccinated [15]), a recent sample of people receiving community‐based hepatitis C treatment in the US who inject drugs (35% susceptible [16]), and two community samples of people who inject drugs recruited in Australia over a decade ago (26.5%–45% susceptible [17, 18]). Just over half the sample accurately reported their immune status to HBV. Participants born in Canada prior to the introduction of universal childhood vaccination programs had higher odds of susceptibility to infection, while those currently in OAT and with a history of hepatitis C infection had lower odds.

Despite recommendations that people who inject drugs receive hepatitis B vaccination, a minority of this sample remain susceptible to HBV infection. The gap in coverage may be a result of multilevel barriers this population experiences to non‐emergency healthcare [19]. Service providers have noted that vaccine hesitancy is a key barrier to providing hepatitis B vaccination to this population [7]. Information on specific drivers of hepatitis B vaccine hesitancy is lacking, but multiple studies have investigated COVID‐19 vaccine hesitancy and found that low risk perception of disease, vaccine misinformation, and institutional distrust contribute to lower intention to be vaccinated [20, 21, 22, 23]. The association with birth cohort suggests that coverage will be higher among new cohorts of people who inject drugs. However, the continued role of injecting drug use in HBV transmission in Canada means it is critical to maximise vaccination coverage among those currently injecting drugs and susceptible to infection [24].

Provision of hepatitis B immunisation services at low threshold settings (e.g., needle syringe programs) has improved uptake [25]. Our finding that 40% of participants did not accurately report their HBV status supports previous advice to offer vaccination to all individuals without evidence of immunity [26]. Offering screening in parallel with the first vaccine dose is a cost‐effective strategy to prevent new infections [26] and ensures that existing undiagnosed cases are linked to care [27]. In traditional health settings, a barrier to free hepatitis B vaccination is the requirement to disclose injecting drug use (or other risk factor) which can be associated with stigma and discrimination. In the US, immunisation policy was recently updated to recommend universal adult hepatitis B vaccination, meaning injecting drug use no longer needs to be disclosed, which may improve uptake among this group [28]. Given the gap in coverage despite vaccination policy recommendations, targeted efforts to improve uptake among people who inject drugs are warranted.

Consistent with other studies of vaccine uptake among people who inject drugs [12, 29, 30], OAT was associated with decreased odds of susceptibility to HBV infection. Although we could not distinguish between vaccine‐ and infection‐induced immunity, almost all participants whose serology indicated they were immune to infection reported vaccination rather than infection. OAT is consistently associated with more favourable health outcomes [31], likely in part due to contact with primary health services or community‐based pharmacies. This reiterates the important role low‐threshold services might play in referring people not in OAT to immunisation services. Encouragingly, people with a history of hepatitis C virus infection, who are at risk of more severe negative health outcomes if co‐infected with hepatitis B, had lower odds of susceptibility to HBV infection. We found no evidence that incarceration was associated with lower HBV susceptibility, indicating a missed opportunity for vaccination. Given the overrepresentation of people who inject drugs in carceral settings, as well as high rates of tattooing [32], prisons could consider opt‐out vaccination for non‐immune people entering prison.

### 4.1 Limitations

The primary limitation of this study is that HBsAb titres may decline below the level of detection despite the individual being immune. Therefore, we may have overestimated the proportion of the sample susceptible to infection. Linkage with a vaccine registry, which Quebec introduced in 2019 to capture all subsequent vaccinations and any previous electronically recorded vaccinations, could mitigate this issue and provide a mechanism to routinely monitor coverage. This was a service‐engaged sample recruited in a single city and may not be representative of the broader population of people in Canada who inject drugs.

## 5 Conclusion

Despite the inclusion of people who inject drugs in Canadian hepatitis B vaccination policy recommendations, approximately one‐third of this sample were susceptible to HBV infection. Given evidence for continued transmission of HBV among this population, improving vaccination coverage is critical to Canada achieving the World Health Organization hepatitis elimination goals by 2030. People who inject drugs and were born prior to the introduction of universal childhood vaccination programs or who are not receiving OAT are particular subpopulations who warrant targeted immunisation delivery.

## Author Contributions

All authors were involved in the conceptualisation and design of the study. O.P. conducted the analysis and drafted the manuscript. All authors were involved in critical review and editing of the manuscript.

## Conflicts of Interest

S.L. has received advisory board fees from Gilead Sciences. J.B. has received advisory board fees from Gilead Sciences, AbbVie and Cepheid, and research grant from Gilead Sciences, outside the scope of this work. The remaining authors have no competing interests to declare.

## Data Availability

Research data are not shared.

## Appendix A

**TABLE A1:** Sociodemographic characteristics of HEPCO participants interviewed since November 2022.

	Included in HBV analysis, *n* (row %)	Excluded from HBV analysis, *n* (row %)	*p*
Total	384 (100)	34 (100)	—
Eligibility for Canadian universal childhood vaccination program			
Eligible	72 (18.8)	6 (17.6)	0.952
Ineligible, born in Canada prior to 1985	286 (74.5)	25 (73.5)	
Ineligible, born outside of Canada	23 (6.0)	1 (2.9)	
Missing	3 (0.8)	2 (5.9)	
Gender			
Man/male	316 (82.3)	22 (64.7)	0.032
Woman/female	57 (14.8)	9 (26.5)	
Non‐binary, queer or other gender identity	7 (1.8)	2 (5.9)	
Missing	4 (1.0)	1 (2.9)	
Ethnicity			
White	335 (87.2)	30 (88.2)	> 0.999
Aboriginal, Inuit or Métis	16 (4.2)	1 (2.9)	
Other	33 (8.6)	3 (8.8)	
Missing	0 (0)	30 (88.2)	
Housing^a^			
Stable	205 (53.4)	17 (50.0)	0.955
Unstable	24 (6.2)	2 (5.9)	
Street or shelter	154 (40.1)	14 (41.2)	
Missing	1 (0.3)	1 (2.9)	
Incarceration			
Never	106 (27.6)	8 (23.5)	0.910
Yes, > 3 months ago	242 (63.0)	19 (55.9)	
Yes, ≤ 3 months ago	33 (8.6)	3 (8.8)	
Missing	3 (0.8)	4 (11.8)	
Opioid agonist treatment^a^			
No	210 (54.7)	13 (38.2)	0.197
Yes	171 (44.5)	18 (52.9)	
Missing	3 (0.8)	3 (8.8)	
Hepatitis C antibody status			
Negative	155 (40.4)	6 (17.6)	0.202
Positive	222 (57.8)	17 (50.0)	
Missing	7 (1.8)	11 (32.4)	
Duration of injecting drug use			
< 5 years	52 (13.5)	3 (8.8)	0.599
≥ 5 years	330 (85.9)	31 (91.2)	
Missing	2 (0.5)	0 (0)	

^a^ In the past 3 months.

## References

[1] A. Syangbo , M. Hickman , S. Colledge‐Frisby , et al., “Associations Between the Prevalence of Chronic Hepatitis B Among People Who Inject Drugs and Country‐Level Characteristics: An Ecological Analysis,” Drug and Alcohol Review 42, no. 3 (2023): 569–581. 36600489 10.1111/dar.13595 PMC10728688

[2] L. Degenhardt , P. Webb , S. Colledge‐Frisby , et al., “Epidemiology of Injecting Drug Use, Prevalence of Injecting‐Related Harm, and Exposure to Behavioural and Environmental Risks Among People Who Inject Drugs: A Systematic Review,” Lancet Global Health 11, no. 5 (2023): e659–e672, 10.1016/S2214-109X(23)00057-8. 36996857 PMC12580156

[3] C. J. Chu and S. D. Lee , “Hepatitis B Virus/Hepatitis C Virus Coinfection: Epidemiology, Clinical Features, Viral Interactions and Treatment,” Journal of Gastroenterology and Hepatology 23, no. 4 (2008): 512–520. 18397482 10.1111/j.1440-1746.2008.05384.x

[4] Public Health Agency of Canada , “Hepatitis B Vaccines: Canadian Immunization Guide: Government of Canada.” 2023, https://www.canada.ca/en/public‐health/services/publications/healthy‐living/canadian‐immunization‐guide‐part‐4‐active‐vaccines/page‐7‐hepatitis‐b‐vaccine.html.

[5] Québec Ministère de la Santé et des Services Sociaux , “HB : Vaccin Contre L'hépatite B 2023.” https://msss.gouv.qc.ca/professionnels/vaccination/piq‐vaccins/hb‐vaccin‐contre‐l‐hepatite‐b/.

[6] O. Price , R. Swanton , J. Grebely , et al., “Vaccination Coverage Among People Who Inject Drugs: A Systematic Review,” International Journal on Drug Policy 127 (2024): 104382. 38503233 10.1016/j.drugpo.2024.104382

[7] B. Zovich , C. Freeland , H. Moore , et al., “Identifying Barriers to Hepatitis B and Delta Screening, Prevention, and Linkage to Care Among People Who Use Drugs in Philadelphia, Pennsylvania, USA,” Harm Reduction Journal 21, no. 1 (2024): 199. 39548537 10.1186/s12954-024-01117-4 PMC11566396

[8] M. Binka , Z. A. Butt , S. Wong , et al., “Differing Profiles of People Diagnosed With Acute and Chronic Hepatitis B Virus Infection in British Columbia, Canada,” World Journal of Gastroenterology 24, no. 11 (2018): 1216–1227, 10.3748/wjg.v24.i11.1216. 29568202 PMC5859224

[9] J. Cox , P. De , C. Morissette , et al., “Low Perceived Benefits and Self‐Efficacy Are Associated With Hepatitis C Virus (HCV) Infection‐Related Risk Among Injection Drug Users,” Social Science and Medicine 66, no. 2 (2008): 211–220. 17920741 10.1016/j.socscimed.2007.08.022 PMC2929253

[10] L. Topp , C. Day , G. J. Dore , and L. Maher , “Poor Criterion Validity of Self‐Reported Hepatitis B Infection and Vaccination Status Among Injecting Drug Users: A Review,” Drug and Alcohol Review 28, no. 6 (2009): 669–675. 19930022 10.1111/j.1465-3362.2009.00060.x

[11] N. Minoyan , A. A. Artenie , G. Zang , D. Jutras‐Aswad , M. E. Turcotte , and J. Bruneau , “Harm Reduction Coverage and Hepatitis C Incidence: Findings From a Cohort of People Who Inject Drugs,” American Journal of Preventive Medicine 58, no. 6 (2020): 845–853. 32444003 10.1016/j.amepre.2020.01.024

[12] O. Price , P. Dietze , L. Maher , et al., “High COVID‐19 Vaccine Uptake Following Initial Hesitancy Among People in Australia Who Inject Drugs,” Vaccine 42, no. 11 (2024): 2877–2885. 38519346 10.1016/j.vaccine.2024.03.051

[13] F. Lugoboni , G. Quaglio , P. Civitelli , and P. Mezzelani , “Bloodborne Viral Hepatitis Infections Among Drug Users: The Role of Vaccination,” International Journal of Environmental Research and Public Health 6, no. 1 (2009): 400–413. 19440291 10.3390/ijerph6010400 PMC2672321

[14] N. Vicente‐Alcalde , E. Ruescas‐Escolano , Z. B. Harboe , and J. Tuells , “Vaccination Coverage Among Prisoners: A Systematic Review,” International Journal of Environmental Research and Public Health 17, no. 20 (2020): 7589. 33086513 10.3390/ijerph17207589 PMC7589151

[15] Government of Canada , “Adult National Immunization Coverage Survey (aNICS): 2023 Results 2024.” Available from: https://www.canada.ca/en/public‐health/services/immunization‐vaccines/vaccination‐coverage/adult‐national‐immunization‐coverage‐survey‐2023‐results.html.

[16] C. Campusano , R. Kanner , C. McDonell , M. Morris , M. Duarte , and J. C. Price , “High Prevalence of Hepatitis B Virus Susceptibility Among Persons Undergoing Community‐Based Hepatitis C Virus Treatment,” Harm Reduction Journal 21, no. 1 (2024): 25. 38281942 10.1186/s12954-024-00942-x PMC10823661

[17] R. J. Winter , P. M. Dietze , M. Gouillou , M. E. Hellard , P. Robinson , and C. K. Aitken , “Hepatitis B Virus Exposure and Vaccination in a Cohort of People Who Inject Drugs: What has Been the Impact of Targeted Free Vaccination?,” Journal of Gastroenterology and Hepatology 28, no. 2 (2013): 314–322. 23190264 10.1111/jgh.12063

[18] B. White , G. J. Dore , A. Lloyd , W. Rawlinson , and L. Maher , “Ongoing Susceptibility to Hepatitis B Virus Infection Among People Who Inject Drugs in Sydney,” Journal of the Australian and New Zealand Journal of Public Health 36, no. 4 (2012): 351–356, 10.1111/j.1753-6405.2012.00881.x.

[19] D. Motavalli , J. L. Taylor , E. Childs , et al., ““Health Is on the Back Burner:” Multilevel Barriers and Facilitators to Primary Care Among People Who Inject Drugs,” Journal of General Internal Medicine 36, no. 1 (2021): 129–137. 32918199 10.1007/s11606-020-06201-6 PMC7858998

[20] S. A. Strathdee , D. Abramovitz , A. Harvey‐Vera , et al., “Correlates of Coronavirus Disease 2019 (COVID‐19) Vaccine Hesitancy Among People Who Inject Drugs in the San Diego‐Tijuana Border Region,” Clinical Infectious Diseases 75, no. 1 (2021): e726–e733, 10.1093/cid/ciab975. PMC8690110 35024825

[21] O. Price , L. Maher , P. M. Dietze , et al., “COVID‐19 Vaccine Attitudes and Facilitators Among People in Australia Who Inject Drugs,” Drug and Alcohol Review 42, no. 5 (2023): 1066–1077. 36802338 10.1111/dar.13621

[22] I. D. Aronson , A. S. Bennett , M. A. Ardouin‐Guerrier , et al., “How Vaccine Ambivalence Can Lead People Who Inject Drugs to Decline COVID‐19 Vaccination and Ways This Can be Addressed: Qualitative Study,” JMIR Formative Research 6, no. 3 (2022): e35066. 35191841 10.2196/35066 PMC8945077

[23] C. C. Cioffi , D. Kosty , S. Nachbar , C. G. Capron , A. M. Mauricio , and H. F. Tavalire , “COVID‐19 Vaccine Deliberation Among People Who Inject Drugs,” Drug and Alcohol Dependence Reports 3 (2022): 100046. 35345466 10.1016/j.dadr.2022.100046 PMC8942572

[24] L. Degenhardt , F. Charlson , J. Stanaway , et al., “Estimating the Burden of Disease Attributable to Injecting Drug Use as a Risk Factor for HIV, Hepatitis C, and Hepatitis B: Findings From the Global Burden of Disease Study 2013,” Lancet Infectious Diseases 16, no. 12 (2016): 1385–1398. 27665254 10.1016/S1473-3099(16)30325-5

[25] D. C. Des Jarlais , D. G. Fisher , J. C. Newman , et al., “Providing Hepatitis B Vaccination to Injection Drug Users: Referral to Health Clinics vs On‐Site Vaccination at a Syringe Exchange Program,” American Journal of Public Health 91, no. 11 (2001): 1791–1792. 11684603 10.2105/ajph.91.11.1791 PMC1446878

[26] Y. Hu , L. E. Grau , G. Scott , et al., “Economic Evaluation of Delivering Hepatitis B Vaccine to Injection Drug Users,” American Journal of Preventive Medicine 35, no. 1 (2008): 25–32. 18541174 10.1016/j.amepre.2008.03.028 PMC2483306

[27] ASHM , “Decision Making In Hepatitis B Sydney, Australia: Australasian Society for HIV Medicine, Viral Hepatitis and Sexual Health Medicine; 2024.” https://ashm.org.au/resources/decision‐making‐in‐hepatitis‐b/.

[28] M. K. Weng , M. Doshani , M. A. Khan , et al., “Universal Hepatitis B Vaccination in Adults Aged 19‐59 Years: Updated Recommendations of the Advisory Committee on Immunization Practices ‐ United States, 2022,” MMWR. Morbidity and Mortality Weekly Report 71, no. 13 (2022): 477–483. 35358162 10.15585/mmwr.mm7113a1 PMC8979596

[29] O. Price , P. Dietze , S. G. Sullivan , C. Salom , and A. Peacock , “Uptake, Barriers and Correlates of Influenza Vaccination Among People Who Inject Drugs in Australia,” Drug and Alcohol Dependence 226 (2021): 108882. 34216866 10.1016/j.drugalcdep.2021.108882

[30] J. Iversen , H. Wand , R. Kemp , et al., “Uptake of COVID‐19 Vaccination Among People Who Inject Drugs,” Harm Reduction Journal 19, no. 1 (2022): 59. 35655217 10.1186/s12954-022-00643-3 PMC9162792

[31] T. Santo, Jr. , B. Clark , M. Hickman , et al., “Association of Opioid Agonist Treatment With All‐Cause Mortality and Specific Causes of Death Among People With Opioid Dependence: A Systematic Review and Meta‐Analysis,” JAMA Psychiatry 78, no. 9 (2021): 979–993. 34076676 10.1001/jamapsychiatry.2021.0976 PMC8173472

[32] B. Moazen , S. Saeedi Moghaddam , M. A. Silbernagl , et al., “Prevalence of Drug Injection, Sexual Activity, Tattooing, and Piercing Among Prison Inmates,” Epidemiologic Reviews 40, no. 1 (2018): 58–69. 29860343 10.1093/epirev/mxy002

